# lncRNA SNHG3 acts as a novel Tumor Suppressor and regulates Tumor Proliferation and Metastasis via AKT/mTOR/ERK pathway in Papillary Thyroid Carcinoma

**DOI:** 10.7150/jca.42070

**Published:** 2020-03-15

**Authors:** Yu Duan, Zhiyong Wang, Lijuan Xu, Li Sun, Hairong Song, Huiqing Yin, Fucheng He

**Affiliations:** 1 Department of Medical Laboratory, The First Affiliated Hospital of Zhengzhou University, Zhengzhou, Henan 450000, China.; 2 Department of Urology, The First Affiliated Hospital of Zhengzhou University, Zhengzhou, Henan 450000, China.

**Keywords:** Papillary thyroid cancer, Long non-coding RNA, SNHG3, mTOR, AZD8055

## Abstract

The incidence of papillary thyroid carcinoma (PTC) has been increased rapidly in recent decades. Long noncoding RNAs (lncRNA) are a class of non-protein-coding transcripts and play critical roles in regulating gene expression and influence biological behaviors of multiple cancers, including PTC. Here, we discovered that lncRNA SNHG3 was significantly downregulated in PTC tissues and cell lines, the expression of SNHG3 was negatively correlated with the TNM stage and poor prognosis of PTC patients. Functional studies illustrated that the depletion of SNHG3 via CRISPR/Cas9 technology promoted the proliferation, migration and invasion abilities of PTC cells. Tumor xenograft models confirmed the tumor-promoting role of silenced SNHG3 *in vivo*. Further mechanistic analyses revealed that knockout of SNHG3 activated the AKT/mTOR/ERK pathway in PTC cell lines and the mTOR inhibitor AZD8055 abrogated the tumor-promoting effect induced by SNHG3 inhibition. Taken together, our findings identified a lncRNA SNHG3 that functions its tumor-suppressor role during PTC development and SNHG3 might serve as a promising candidate for target therapy of PTC.

## Introduction

Thyroid cancer is the most prevalent endocrine malignancy and the fifth most common cancer in women, ranking in ninth place for the global cancer incidence, with approximately 570000 new cases annually around the world 1. Thyroid cancer can be categorized into four groups based on pathology type, papillary thyroid carcinoma (PTC) of which is the most frequently diagnosed type, accounting for 90% cases of thyroid cancers 2. Most patients with PTC have an overall 5-year survival rate above 90%; however, about 15% of patients exhibited poor prognosis owing to the tumor metastasis 3. Although the genomic landscape of PTC has been extensively characterized, the intrinsic-factors that control thyroid cancer development are less well understood. Therefore, identifying the novel targets for thyroid tumorigenesis and progression is of particular interest.

Long noncoding RNAs (lncRNAs) are transcripts with over 200 nucleotides in length without protein-coding capabilities. Although lncRNAs had been regarded as the “desert region” of the genome for many years, recently increasing evidence has disclosed that lncRNAs play critical roles in regulating gene expression at various aspects, including mRNA alternative splicing, protein activities, alteration of protein localization and epigenetic programming 4,5. A variety of lncRNAs are aberrantly expressed in specific cancer types 6. Notably, the underlying mechanisms of lncRNAs contributing to cancer development are diverse 7. They are involved in human malignancies through driving many crucial cancer phenotypes, including cell cycle regulation, survival, immune response and pluripotency in cancer cells 8. For instance, Kawasaki *et al.* identified a lncRNA, MYU, that mediated the induction of CDK6 to promote cell cycle progression in colon cancer cells 9. lncRNA-Hh strengthens cancer stem cell generation in breast cancer via activation of the hedgehog signaling pathway 10.

Small nucleolar host gene 3 (SNHG3) is a novel lncRNA located at 1p36.1. SNHG3 was firstly implicated in Alzheimer's disease (AD) and its up-regulation might be an indicator of a wider dysregulation of translational machinery and ribosome biogenesis during AD neurodegeneration 11. Subsequently, it was revealed to be an oncogene and a biomarker of malignant status or poor prognosis in several types of cancers, including colorectal cancer, ovarian cancer and hepatocellular carcinoma 12-14. However, no studies to date reported its potential tumor suppressor function in cancer.

In the current study, we revealed that SNHG3 was silenced in PTC tissues and cell lines and inhibition of SNHG3 via CRISPR/Cas9 facilitated PTC cell proliferation, migration and invasion. Mechanistic studies demonstrated that SNHG3 promoted PTC malignant progression through AKT/mTOR/ERK signaling pathway. Furthermore, blockade of mTOR by AZD8055 hampered the tumor-promoting effect from the loss of SNHG3. To the best of knowledge, our study, for the first time, highlighted the pivotal role of SNHG3 as a tumor suppressor in PTC tumorigenicity.

## Materials and Methods

### Human PTC samples collection

All samples were obtained from the First Affiliated Hospital of Zhengzhou University between January 2017 and December 2018, who diagnosed with PTC. Histopathologic diagnoses were confirmed by the pathologists and classified based on the 7th American Joint Committee on Cancer (AJCC) staging system. Patients were excluded from study if their thyroid malignancy was not of papillary histology or if they received treatment with radioiodine therapy or chemotherapy before surgery. All patients were provided with written informed consent and were treated with total or near-total thyroidectomy for PTC in surgery. This study procedure was approved by the medical ethics committee of the First Affiliated Hospital of Zhengzhou University.

### Cell culture

Human immortalized thyroid cells Nthy-ori 3-1 and human PTC cell lines BCPAP, KTC-1 and TPC-1 were purchased from the Stem Cell Bank, Chinese Academy of Science (Shanghai, China). All cell lines were maintained in high glucose DMEM (BI, Israel) supplemented with 10% FBS, 100 units/mL of penicillin and 100 μg/ml of streptomycin at 37°C in 5% CO_2_ atmosphere. All cells were confirmed without mycoplasma infection.

### Cytoplasmic and nuclear fractionation

The nuclear and cytoplasmic fractions of cells were isolated by the invent Minute^TM^ Cytoplasmic nuclear separation kit (Invent Biotechnologies) following the protocols of the manufacturer and treated with TRIzol reagent to obtain RNA. RNA levels of SNHG3, U1 and β-actin in the nuclear and cytoplasmic fractions were detected by qPCR. β-actin was as a cytoplasmic control, and U1 was as a nuclear control.

### Real-time PCR

Total RNAs were isolated from tissues or cells using TRIzol reagent (Invitrogen Carlsbad, CA, USA). For quantification, cDNAs were synthesized by Prime Script RT Reagent Kit (Takara, Tokyo, Japan), followed by real-time quantitative PCR using SYBR Premix Ex Taq™ (Takara, Tokyo, Japan). Primer sequences were listed as follows: SNHG3 (Forward, 5′-GGAAATAAAGCTGGGCCTCG-3′ and reverse, 5′- AACAGAGCGACTCCATCTCC-3′), U1 (Forward, 5′- GACGGGAAAAGATTGAGCGG -3′ and reverse, 5′-GCCACGAAGAGAGTCTTGAAGG-3′), β-actin (Forward, 5′- CATGTACGTTGCTATCCAGGC -3′ and reverse, 5′- CTCCTTAATGTCACGCACGAT -3′). Gene expression was normalized to the housekeeping gene β-actin and analyzed according to the relative quantification method (2^-ΔΔCt^).

### CRISPR/Cas9-mediated knockout of SNHG3

For stable knock-out of SNHG3 in cells, a pair of guide RNAs targeting SNHG3 were cloned into lentiCRISPR v2 vectors, sgRNA sequences targeting SNHG3 were listed as follows: (Forward, 5′-CACCGGACGGGCCTGGGCCAGAAG-3′ and reverse, 5′-AAACCTTCTGGCCCAGGCCCGTCC-3′). HEK293T cells were transfected with the plasmids using Lipofectamine 2000 (Invitrogen, Carlsbad, CA, USA) reagents. 48 hours after transfection, viral supernatant was collected, filtered through a 0.45-μm filter, and then added to BCPAP and TPC-1 cells. Stable cell lines were selected using puromycin (2.0 μg/mL) for two weeks and the knock-out efficiency was validated by RT-PCR.

### Western blot

Cells were collected and lysed in RIPA (Yeasen, Shanghai, China) with protease inhibitor cocktail (Sigma-Aldrich, St.Louis, MO, USA). Equivalent protein was loaded onto 10% SDS-PAGE gel and transferred into nitrocellulose membranes (PALL, NewYork, USA). The blots were incubated with specific primary antibodies, followed by secondary antibodies. The proteins were visualized with the Clinx ChemiScope (Clinx Science Instruments, Shanghai, China). The antibodies used for western blot were listed as follows: anti-AKT antibodies (#9272, Cell Signaling Technology), anti-p-AKT antibodies (#4060, Cell Signaling Technology), anti-mTOR antibodies (#2983, Cell Signaling Technology), anti-p-mTOR antibodies (#5536, Cell Signaling Technology), anti-ERK antibodies (#4695, Cell Signaling Technology), anti-p-ERK antibodies (#4370, Cell Signaling Technology), anti-GAPDH antibodies (#5174, Cell Signaling Technology).

### Cell viability assays

For CCK-8 assay, 2 × 10^3^ Cells were seeded on the 96-well plate, and 4 replicate wells were set. 10μl of CCK8 was added into the 96-well plate. After incubating at 37°C for 2 hours, absorbance at the wavelength of 450nm was detected for the growth curve. For EdU assay, 5 × 10^3^ cells were seeded on the 96-well plate and assessed using an EdU Labeling Kit (RiboBio, China) according to the manufacturer's instructions. Briefly, EdU labeling media were added to the plates and they were incubated for 2 hours at 37°C. After treatment with 4% paraformaldehyde and 0.5% Triton X-100, cells were stained with anti-EdU working solution. DAPI was used to label cell nuclei. For colony formation assay, parental or SNHG3-knockout cells (500 cells/well) were plated into the six-well plate and cultured for two weeks. Cells on the plates were fixed with 4% paraformaldehyde and stained with crystal violet.

### Transwell migration and invasion assay

The 24-well transwell chambers (Corning, Carlsbad, CA, USA) were used to measure cell migration (without Matrigel) and invasion (with Matrigel). 1 × 10^5^ cells were resuspended in 200μl serum-free medium and seeded onto the upper chambers. Mitomycin C (Sigma) was used to inhibit cell proliferation. The lower wells were filled with 700μl 1640 or DMEM medium with 20% fetal bovine serum. Incubating at 37°C for 24h (migration) or 48h (invasion), the migrated or invaded cells were fixed in 4% paraformaldehyde for 20min and stained with 0.1% crystal violet for 20min.

### Xenograft mouse model

Five-week-old female BALB/c nude mice were purchased from the Vital River Laboratory Animal Technology and housed under pathogen-free conditions at 22°C in the 12h light/dark cycle. Animal protocols were performed according to the guidelines of Animal Ethics Committee of the First Affiliated Hospital of Zhengzhou University. Briefly, ten mice were randomly divided into scramble and SNHG3-KO group and subcutaneously injected with 5 × 10^6^ BCPAP cells/mouse in 150 µl PBS into their right flanks. Tumor volumes were monitored every week with digital caliper using the following formula: (length × width^2^)/2. Four weeks after injection, the mice were sacrificed and tumors were extracted for further studies.

### Statistical analysis

All statistical analyses were performed by paired two-tailed Student's t-test or Chi-Square test with GraphPad 7.0 Software (La Jolla, CA, USA). Results were displayed as mean ± SD from three independent experiments. P-value <0.05 was considered to be statistically significant.

## Results

### SNHG3 is frequently downregulated in PTC tissues

**Figure 1:**
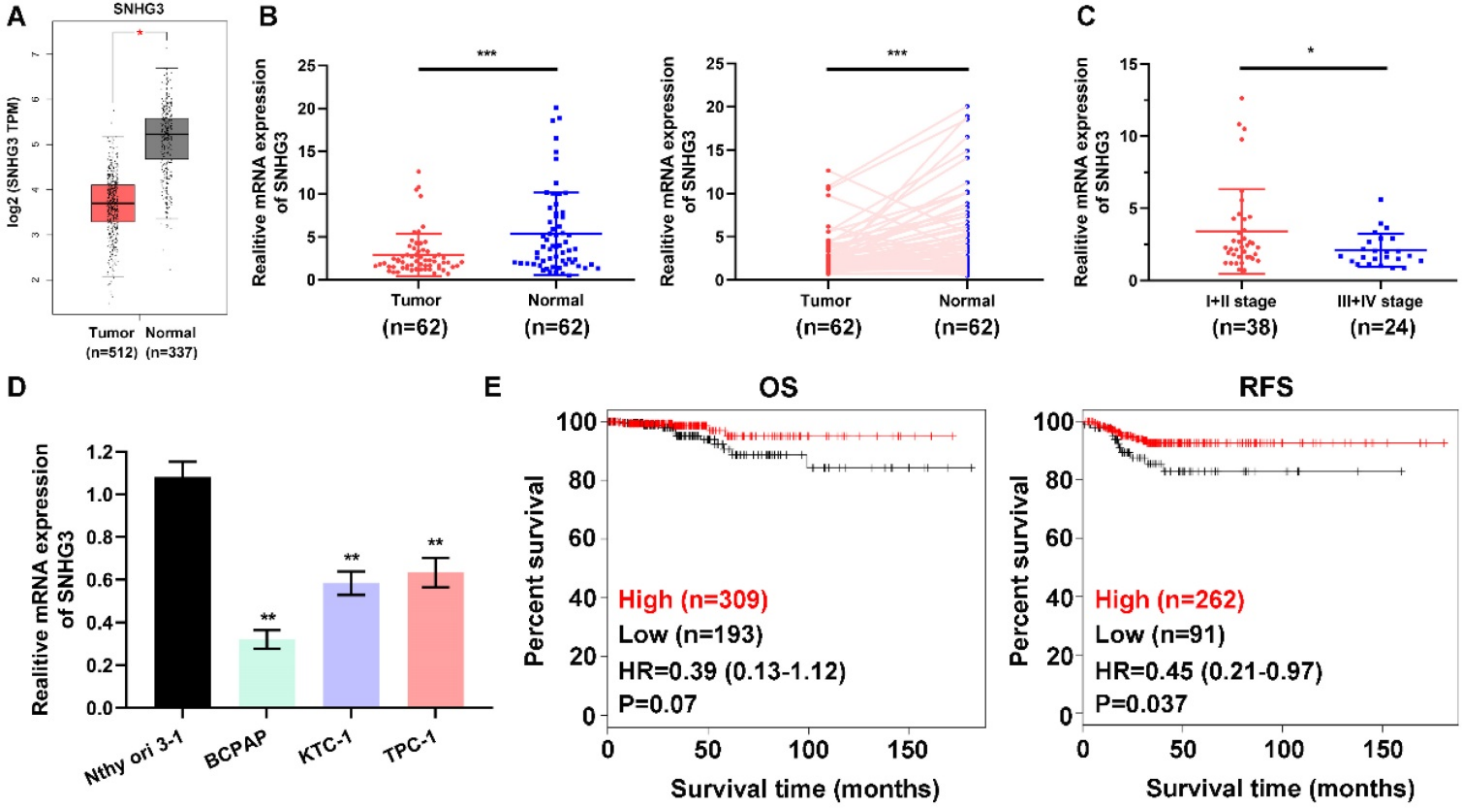
** SNHG3 expression is generally down-regulated in PTC tissues and cells. (A)** The mRNA expression of SNHG3 in PTC patients based on data from GEPIA database.** (B)** The RNA levels of SNHG3 was analyzed in 62 paired PTC and adjacent non-tumor tissues by quantitative RT-PCR. **(C)** Relative expression of SNHG3 in PTC patients with I+II tumor stage compared with patients with the advanced tumor stage. **(D)** SNHG3 expression in immortalized thyroid follicular cells Nthy ori 3-1 and three PTC cell lines was evaluated by RT-PCR. **(E)** Kaplan-Meier survival analysis for overall survival (OS) and recurrence-free survival (RFS) of PTC patients with high vs. low expression of SNHG3 in the TCGA cohort. RT-PCR results were presented as mean ± SD. *P < 0.05, **P < 0.01, ***P < 0.001.

To address the role of SNHG3 in PTC carcinogenesis, we firstly evaluated the SNHG3 expression in the GEPIA platform (http://gepia.cancer-pku.cn/index.html) and the GEO datasets, the bioinformatics analysis results suggested that SNHG3 showed relatively low expression in PTC tissues versus normal thyroid tissues (Fig. 1A and Supplementary Fig. 1A). Next, we performed RT-PCR to examine the SNHG3 level in a cohort of 62 paired human PTC specimens and their matched normal thyroid tissues to validate the data-mining findings. We found that 38/62 (61.3%) of the PTC patients exhibited more than a 1.5-fold decrease of SNHG3 expression in PTC tissues than matched normal thyroid tissues (Table 1 and Fig. 1B). Notably, the deficient level of SNHG3 expression was closely related to the advanced TNM stage of PTC patients (Fig. 1C). Consistently, SNHG3 expression was also lower in three PTC cell lines than normal thyroid follicular cells (Fig. 1D). Moreover, by analyzing the Kaplan-Meier Plotter dataset, low SNHG3 expression was found to be significantly associated with poor clinical outcomes, especially the recurrence-free survival (RFS) in PTC patients (Fig. 1E). Taken together, our results indicated that the decreased expression of SNHG3 might act as a tumor suppressor role and predict poor prognosis in PTC patients.

### Loss of SNHG3 promotes the proliferation and migration abilities of PTC cells

**Figure 2:**
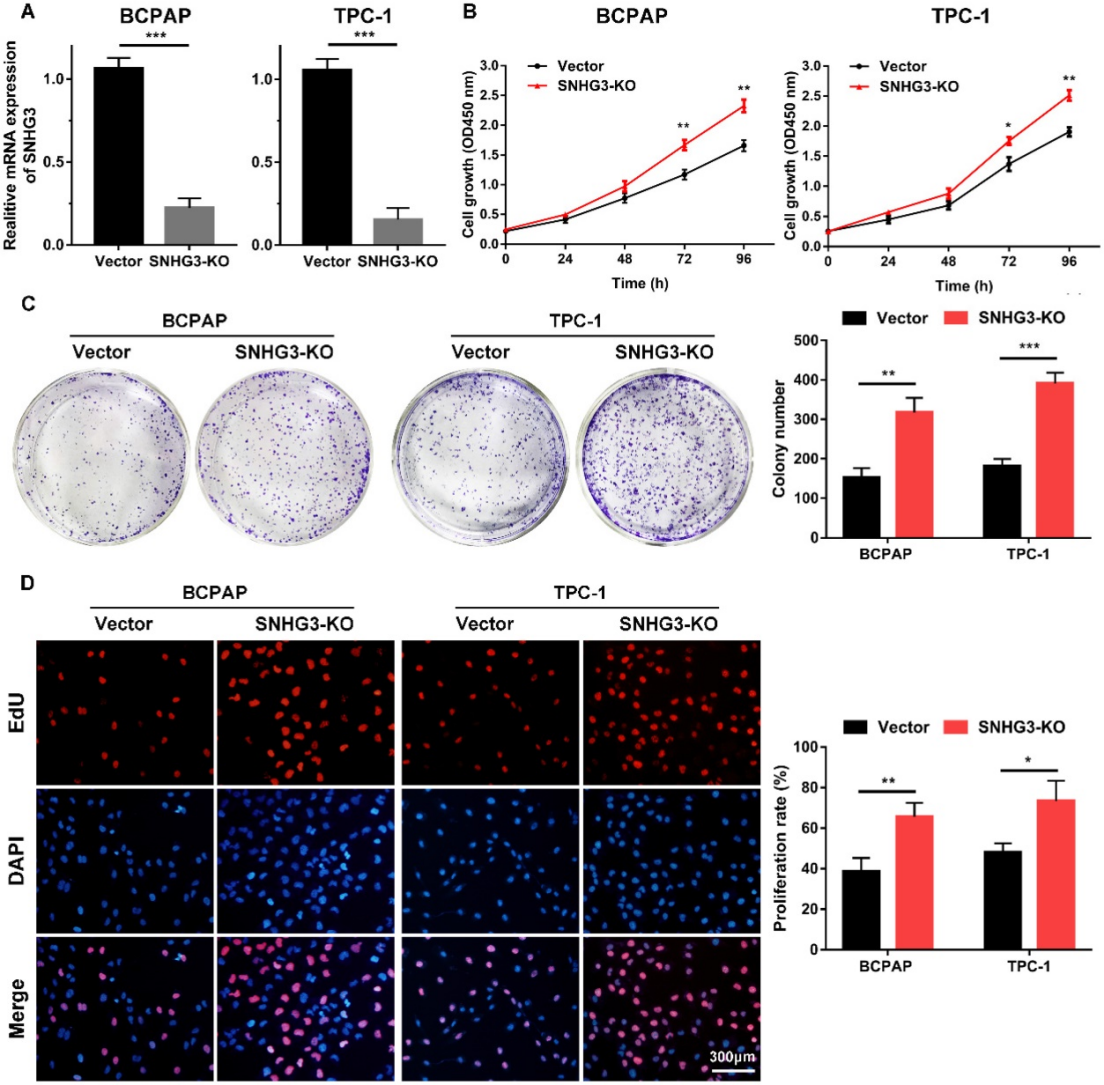
** SNHG3 deficiency promotes PTC cells proliferation *in vitro.* (A)** RT-PCR confirmed the knockout efficiency of SNHG3 by CRISPR/Cas9 technology in the BCPAP and TPC-1 cells. **(B)** CCK-8 assays were performed to verify the functional role of SNHG3 in BCPAP and TPC-1 cell viability. **(C)** Representative graphs and quantification results of colonies in colony formation assay. **(D)** Knock-out of SNHG3 accelerated cell proliferation in PTC cell lines as determined by EdU assays. Error bars represent means ± SD from three independent experiments. *P < 0.05, **P < 0.01, ***P < 0.001.

**Figure 3:**
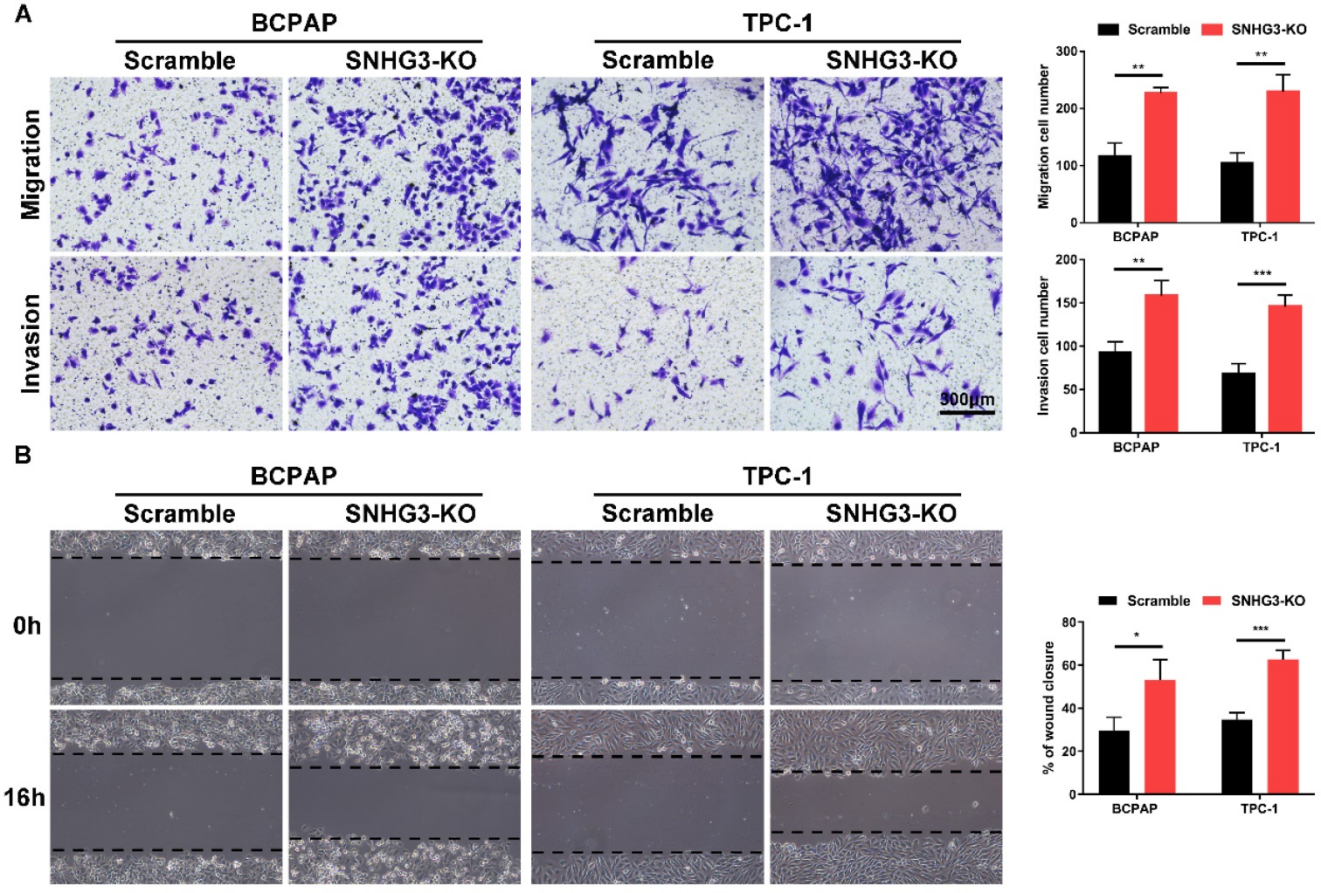
** Inhibition of SNHG3 regulates PTC cells migration and invasion *in vitro.* (A)** Transwell assays with or without matrigel showed that SNHG3 silencing promoted PTC cells migration (upper panel) and invasion (lower panel). **(B)** Wound-healing experiments confirmed the effect of SNHG3 on migration abilities in BCPAP and TPC-1 cells. Representative images (left panel) and quantification analyses (right panel) are shown. Data indicate the mean ± SD based on three independent experiments. *P < 0.05, **P < 0.01, ***P < 0.001.

The strong association of SHNG3 expression with the advanced tumor stage and poor prognosis of PTC patients prompted us to investigate whether SNHG3 was required for PTC cell growth and metastasis. Therefore, we generated SNHG3-knockout cell lines by CRISPR/Cas9 technology in BCPAP (low level of SHNG3) and TPC-1 (high level of SHNG3) background to inhibit the endogenous RNA level of SNHG3. The silencing of SNHG3 was confirmed by qRT-PCR (Fig. 2A). Moreover, through a cytoplasmic and nuclear RNA isolation assay, we found that over 90% of SNHG3 distributed in the nucleus of both BCPAP and TPC1 cells (Supplementary Fig. 2A). We further analyzed the impact of SNHG3 on the growth of PTC cells by CCK8, EdU incorporation experiment and colony formation assays. SNHG3 deficiency obviously accelerated the cell proliferation of PTC cells, as judged by CCK-8 growth curves (Fig. 2B). As shown in Fig. 2C, knock-out of SNHG3 displayed larger and more colonies compared with the control group. Besides, the results from the EdU test indicated that inhibition of SNHG3 enhanced the viability of PTC cells (Fig. 2D). These results supported the notion that SNHG3 regulated PTC cell proliferation *in vitro*. Next, we performed matrigel-coated and uncoated transwell assays to determine the effect of SNHG3 on the metastasis of PTC cells. The transwell results showed that depletion of SNHG3 remarkably elevated the invasive and migratory capabilities of PTC cells, as evidenced by an increased number of migrated and invaded cells in SNHG3-KO group of BCPAP and TPC-1 cells (Fig. 3A). Wound-healing assays also confirmed that knock-out of SNHG3 promoted the metastatic abilities of PTC cells *in vitro* (Fig. 3B). Therefore, the above findings illustrate that SNHG3 functions as a tumor suppressor during the development and progression of PTC.

### Silencing of SNHG3 activates the AKT/mTOR/ERK pathway in PTC cell lines

**Figure 4:**
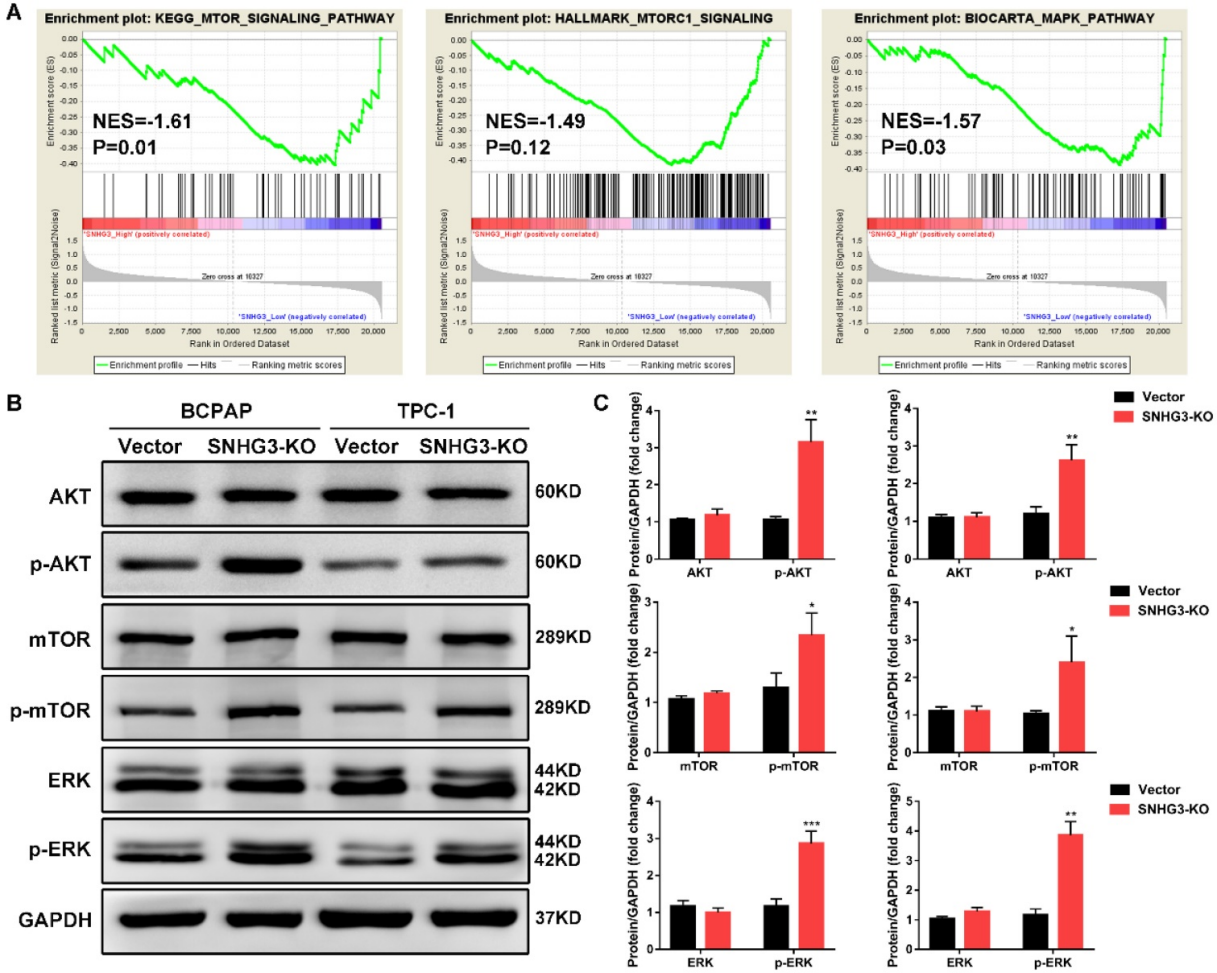
** Bioinformatic analysis reveals that SNHG3 was involved in the AKT/mTOR/ERK pathway. (A)** GSEA analysis comparing the SNHG3-high (red) and low (blue) expression subgroups of PTC patients in the TCGA dataset. Enrichment plots indicate hallmark gene sets enriched in SNHG3-low PTC patients. **(B)** Western blot analysis for mTOR, mTOR (Ser2481), AKT, AKT (Ser473), ERK and ERK (Thr202/Tyr204) in SNHG3 knockout and control group of BCPAP and TPC-1 cells. **(C)** The densitometric analysis of the western blot bands shown in Fig. 4B. The gray values are expressed as the mean ± SD from three independent experiments. *P < 0.05, **P < 0.01, ***P < 0.001.

In order to elucidate the molecular mechanisms underlying the inhibition of SNHG3-induced malignant phenotypes, we applied RNA-seq expression profiles from TCGA THCA dataset to search for differentially expressed genes and enriched pathways between PTC patients with SNHG3-high and SHNG3 low expression. Gene set enrichment analysis (GSEA) revealed that the activation of mTOR signaling pathway and MAPK pathway were strongly related to the low expression of SNHG3 in PTC patients (Fig. 4A). We then performed western blot to detect the crucial protein kinases associated with these pathways. As displayed in Fig. 4B, inactivation of SNHG3 markedly led to the elevation of phosphorylated AKT, mTOR and ERK without affecting the total protein levels of these genes in both BCPAP and TPC-1 cells (Fig. 4B and C).

### mTOR inhibitor AZD8055 abrogates the tumor-promoting effect of SNHG3 inhibition

**Figure 5:**
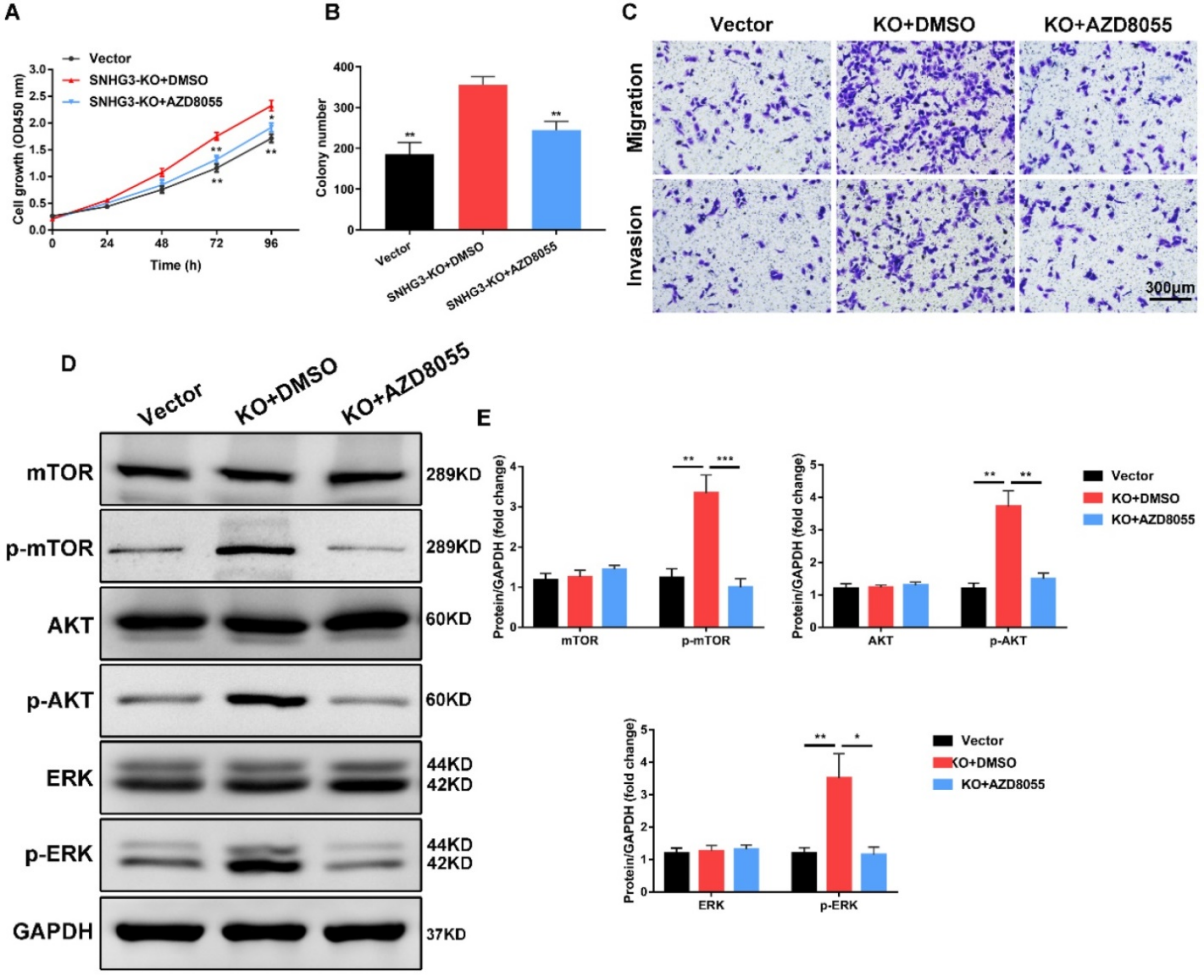
** mTOR inhibitor AZD8055 impedes SNHG3-mediated PTC cell growth, migration and invasion. (A)** BCPAP cells from control and SNHG3-KO group were treated with DMSO or 500 nM AZD8055 for 48h and the cell viability was detected by CCK-8 assays.** (B)** The quantification of colonies per well was counted and analyzed from PTC cells in the presence or absence of AZD8055. **(C)** Transwell experiments showing the effects of SNHG3 on BCPAP cell migration and invasion following treatment with AZD8055 for 48h. **(D)** Western blotting analysis of the protein levels of mTOR, AKT, ERK and their phosphorylation forms in BCPAP control and knockout cells with AZD8055 for 48h. **(E)** The densitometric analysis results of the western blot bands shown in Fig. 5D. Error bars represent three independent experiments. *P < 0.05, **P < 0.01, ***P < 0.001.

To further investigate the effect of mTOR pathway in SNHG3 participated in PTC proliferation and metastasis, we introduced the mTOR inhibitor AZD8055 (Selleckchem) to conduct a series of functional rescue assays. Strikingly, treating BCPAP cells with AZD8055 prominently reversed the growth-promoting effects in PTC cells induced by the stable knock-out of SNHG3, as manifested by CCK-8 and colony-formation experiments (Fig. 5A and B). Meanwhile, transwell migration and invasion assays showed that mTOR silencing by AZD8055 was sufficient to compromise the metastatic abilities compared to SNHG3-KO cells treated with DMSO (Fig. 5C and Supplementary Fig. 3A). We carried out western blot to determine the involvement of these crucial protein during this process. As expected, the results showed that SNHG3 deficiency followed by mTOR inhibitor AZD8055 alleviated the expression of p-mTOR, p-AKT and p-ERK rather than total level of mTOR, AKT and ERK (Fig. 5D and E). Collectively, these results demonstrate that SNHG3 promotes the growth and metastasis of PTC cells partly through the activation of AKT/mTOR/ERK pathway.

### Knock-out of SNHG3 expedites tumor growth *in vivo*

**Figure 6:**
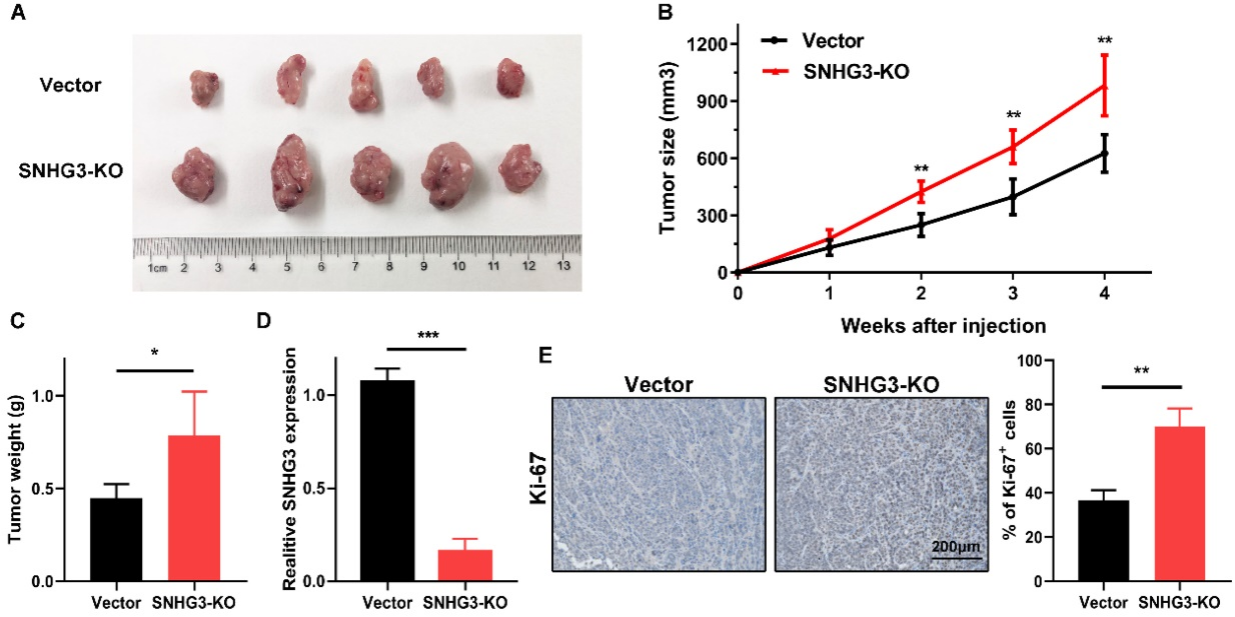
** Knockout of SNHG3 contributes to PTC tumourigenicity *in vivo*. (A)** Representative pictures of tumors formed in nude mouse models after injection of BCPAP cells stably transfected with vector or SNHG3-knockout. **(B)** Growth curve of the tumor volumes was measured every seven days in SNHG3-KO and vector group.** (C)** The average tumor weights were quantified from nude mice in each group. (n=5). **(D)** RT-PCR analysis of the expression of SNHG3 in subcutaneous tumor tissues. **(E)** Representative images of immunohistochemistry staining for Ki-67 and quantification of Ki-67^+^ cells in the indicated subcutaneous xenograft tumors. Error bars represent the mean ± SD. *P < 0.05, **P < 0.01, ***P < 0.001.

To further verify the *in vitro* findings, we used a xenograft model to test the tumor-suppressive role of SNHG3 *in vivo*. An equal amount of control and SNHG3-KO cells were subcutaneously injected into ten BALB/c nude mice. After observing for 4 weeks, we noticed that, compared with the vector group, the mean tumor volume and weight of the SNHG3-KO group were obviously higher, indicating that loss of SNHG3 facilitated the tumor growth *in vivo* (Fig. 6A-C). We also performed the RT-PCR in the xenografts from these two groups and the results verified the low level of SNHG3 expression in SNHG3-KO group (Fig. 6D). Additionally, immunohistochemistry staining showed that SNHG3-KO group displayed a higher positive rate of Ki-67 (Fig. 6E).

## Discussion

Researchers have focused for years to investigate the pathogenesis underlying lncRNAs mediated tumorigenesis. Emerging evidence showed that numerous lncRNAs are critical regulators of metastasis, differentiation, and metabolism of multiple human cancers 15,16. Several lncRNAs have been reported to be involved in PTC tumorigenesis and progression. Yuan *et al.* identified a lncRNA, HOTTIP, that is upregulated in human PTC tissues and associated with growth arrest 17. Zhu* et al.* described the oncogenetic role of lncRNA HOTAIR in PTC and the association between its single nucleotide polymorphisms (SNPs) and PTC risk 18. LncRNA PTCSC3 was significantly suppressed in PTC tumor tissue and it could affect PTC predisposition and carcinogenesis through downregulating the MMP-9 and VEGF expression via the S100A4 pathway 19. LncRNA PVT1 regulated cell growth and cell cycle by functioning as a competing endogenous RNA (ceRNA) to regulate IGF-1 expression by sponging miR-30a 20. Likewise, lncRNA MIAT promoted PTC cell proliferation and migration through sponging miR-212 21. Here, our RT-PCR results confirmed that SNHG3 was markedly silenced in PTC tissues and the expression of SNHG3 was closely related to the TNM stage of PTC patients. Besides, our study indicated that SNHG3 might serve as a novel diagnosis and prognostic biomarker for PTC patients. The above findings emphasized the important clinical value of SNHG3 in PTC patients.

SNHG3 is a novel lncRNA potentially associated with various cancers, including lung adenocarcinoma, ovarian cancer, osteosarcoma, renal cell carcinoma, colorectal cancer and some other cancers 12,13,22-24. SNHG3 promoted gastric cancer progression through regulating neighboring MED18 gene methylation in collaboration with EZH2 25. In osteosarcoma cells, overexpression of SNHG3 increased cell migration and invasion by sponging miR-151a-3p, thereby elevating the expression of its target gene RABB2A 26. To the best of our knowledge, there were no reports regarding the expression or function of SNHG3 in PTC. Consequently, we focused on the potential involvement of SNHG3 in PTC. We further demonstrated SNHG3-deficiency by CRISPR/Cas9 significantly promoted PTC cell viability and proliferation. Besides, we validated the contributions of SNHG3 in tumor metastatic process. SNHG3-silencing strengthened cell migration and invasion abilities as indicated by transwell and wound-healing assays. These phenotypes were further confirmed using *in vivo* xenograft mice model, in which knockout of SNHG3 contributed to PTC tumorigenesis. LncRNAs are known to participate in tumorigenesis either in a pro-tumor or anti-tumor manner 27,28. However, most researches identified SNHG3 as an oncogene in various cancers. Our study, for the first time, demonstrated the anti-tumor effects of SNHG3 in PTC.

Identifying the subcellular localization of lncRNAs could provide the avenue for understanding its underlying molecular mechanism. Through cytoplasmic and nuclear fractionation assays, the results showed that SNHG3 was mainly expressed in the nucleus of PTC cells. Therefore, we postulated that SNHG3 might perform its tumorigenic role at the transcriptional, translational or post-translation level. In order to clarify the potential mechanism by which SNHG3-silencing promotes PTC cell proliferation and invasion, GSEA analysis was performed and uncovered the activation of AKT/mTOR and MAPK/ERK signaling pathway was strongly associated with the low expression of SNHG3 in TCGA PTC patients. As expected, western blot verified that inactivation of SNHG3 distinctly led to the elevation of phosphorylated AKT, mTOR and ERK, indicating that SNHG3-silencing triggered tumor progression in PTC by modulating AKT/mTOR/ERK signaling pathway. This was confirmed by mTOR inhibitor AZD8055, which fully impedes SNHG3-mediated PTC cell growth and metastasis. AZD8055 is a first-in-class orally available ATP-competitive inhibitor of mTOR kinase activity, which selectively blocks phosphorylation of mTOR substrates, including p70S6K (S6K) and 4E-BP1 29. Previous report confirmed that AZD8055 exhibited an obvious tumor-inhibition effect in *HRAS* mutant or *BRAF* mutant PTC cells induced xenograft models 30. Considered as a thyroid oncogenic pathway, AKT/mTOR pathway is mostly hyperactivated in PTC, particularly in poorly differentiated thyroid cancer 31. Apart from the AKT/mTOR pathway, MAPK/ERK pathway is also an important intracellular signaling pathway through a p53-dependent regulation of cell cycle 32. MAPK signaling pathway transmits growth signals from the cell membrane to the cell nucleus and plays a crucial role in promoting PTC cell proliferation and survival 33.

In conclusion, our study demonstrated SNHG3-deficiency could promote PTC cell proliferation, migration, and invasion; and contribute to PTC tumorigenesis both* in vitro* and *in vivo* through activation of AKT/mTOR/ERK pathway. Noticeably, our study for the first time uncovered the anti-tumor properties of SNHG3 in cancer and provided novel insights into the mechanisms by which SNHG3 exerts its tumor suppressor function in PTC.

## Supplementary Material

Supplementary figures.

## Figures and Tables

**Table 1:** Correlation between mRNA expression of SNHG3 and clinical characteristics in 62 PTC patients

Variables	No. of patients	SNHG3 expression		Chi-Square	*P*-value
		High group	Low group		
Gender					
Male	23	9	14	0.0027	0.9583
Female	39	15	24		
Age					
<45	29	11	18	0.0139	0.9061
≥45	33	13	20		
T stage					
T1	36	16	20	1.190	0.2753
T2+T3+T4	26	8	18		
N stage					
N0	28	9	19	0.9280	0.3354
N1a+N1b	34	15	19		
TNM stage					
I+II	38	19	19	5.274	0.0216^*^
III+IV	24	5	19		
Multifocality					
Yes	24	11	13	0.8376	0.3601
No	38	13	25		

*P < 0.05.
