# Supplementary material for: lncRNA SNHG3 acts as a novel Tumor Suppressor and regulates Tumor Proliferation and Metastasis via AKT/mTOR/ERK pathway in Papillary Thyroid Carcinoma
Source: J Cancer. 2020 Mar 15;11(12):3492–501. doi: 10.7150/jca.42070 (PMC7150443; doi:10.7150/jca.42070)
Supplement: Supplementary file 1 — Supplementary figures. [file jcav11p3492s1.pdf]

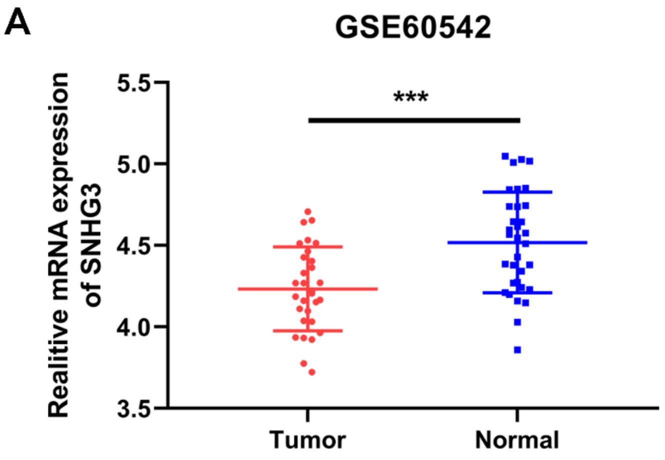

Supplementary Figure 1. (A) Bioinformatics analysis of SLC34A2 mRNA expression on the basis of the GSE60542 confirmed the silenced expression of SNHG3 in PTC tissues.

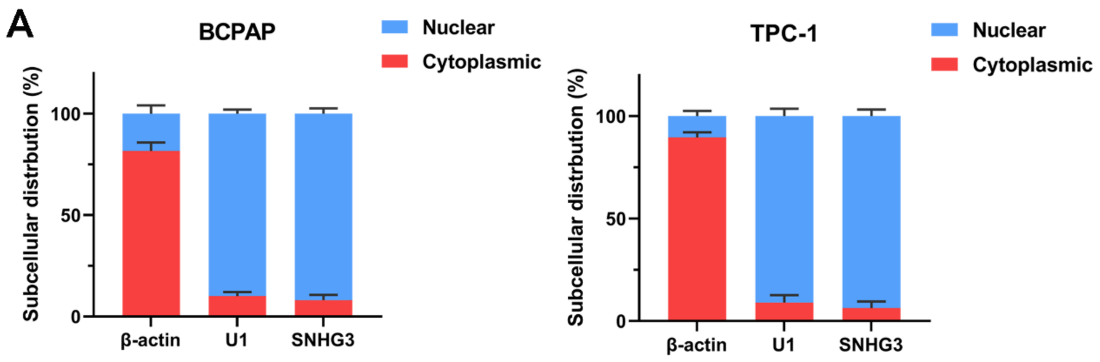

Supplementary Figure 2. (A) The cellular distribution of SNHG3 in BCPAP and TPC-1 cells. β-actin was used as the cytoplasmic internal control and U1 served as the nuclear internal control.

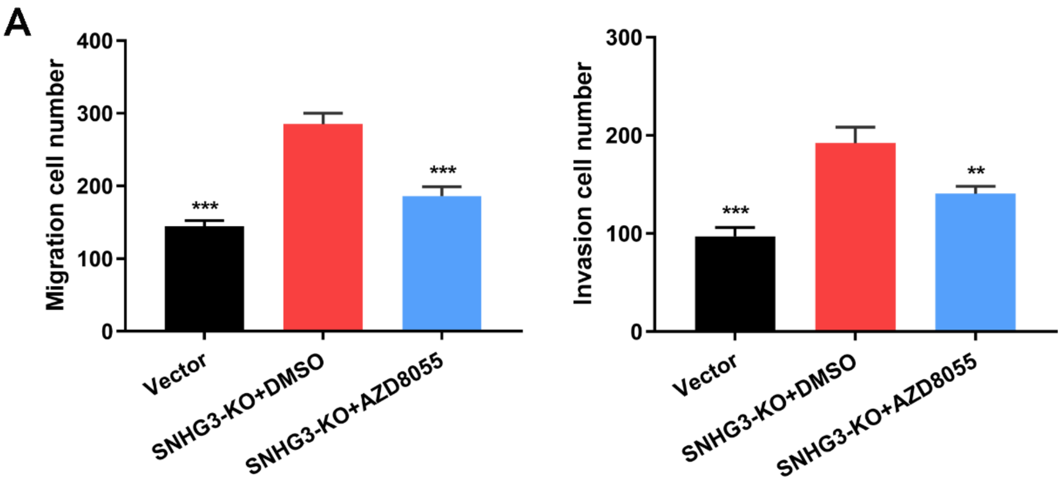

Supplementary Figure 3. (A) The quantification results of transwell migration and invasion assays in control group and SNHG3-KO group treated with DMSO or AZD8055. **P < 0.01, ***P < 0.001.
